# Epicardial fat volume is associated with preexisting atrioventricular conduction abnormalities and increased pacemaker implantation rate in patients undergoing transcatheter aortic valve implantation

**DOI:** 10.1007/s10554-021-02502-x

**Published:** 2021-12-26

**Authors:** Maren Weferling, Andreas Rolf, Ulrich Fischer-Rasokat, Christoph Liebetrau, Matthias Renker, Yeoung-Hoon Choi, Christian W. Hamm, Damini Dey, Won-Keun Kim

**Affiliations:** 1 Department of Cardiology, Kerckhoff Heart and Thorax Center, Benekestr. 2-8, 61231 Bad Nauheim, Germany; 2 German Centre for Cardiovascular Research (DZHK), Partner Site RheinMain, Frankfurt, Germany; 3 Department of Cardiology, University Hospital of Giessen, Giessen, Germany; 4 Cardioangiological Center Bethanien (CCB), Department of Cardiology, Agaplesion Bethanien Hospital, Frankfurt, Germany; 5 Department of Cardiac Surgery, Kerckhoff Heart and Thorax Center, Bad Nauheim, Germany; 6 Biomedical Imaging Research Institute, Cedars-Sinai Medical Center, 8700 Beverly Blvd, Taper A238, Los Angeles, CA 90048 USA

**Keywords:** Epicardial fat tissue, Conduction disorders, Aortic valve stenosis

## Abstract

Epicardial fat tissue (EFT) is a highly metabolically active fat depot surrounding the heart and coronary arteries that is related to early atherosclerosis and adverse cardiac events. We aimed to investigate the relationship between the amount of EFT and preexisting cardiac conduction abnormalities (CCAs) and the need for new postinterventional pacemaker in patients with severe aortic stenosis planned for transcatheter aortic valve implantation (TAVI). A total of 560 consecutive patients (54% female) scheduled for TAVI were included in this retrospective study. EFT volume was measured via a fully automated artificial intelligence software (QFAT) using computed tomography (CT) performed before TAVI. Baseline CCAs [first-degree atrioventricular (AV) block, right bundle branch block (RBBB), and left bundle branch block (LBBB)] were diagnosed according to 12-lead ECG before TAVI. Aortic valve calcification was determined by the Agatston score assessed in the pre-TAVI CT. The median EFT volume was 129.5 ml [IQR 94–170]. Baseline first-degree AV block was present in 17%, RBBB in 10.4%, and LBBB in 10.2% of the overall cohort. In adjusted logistic regression analysis, higher EFT volume was associated with first-degree AV block (OR 1.006 [95% CI 1.002–1.010]; p = 0.006) and the need for new pacemaker implantation after TAVI (OR 1.005 [95% CI 1.0–1.01]; p = 0.035) but not with the presence of RBBB or LBBB. EFT volume did not correlate with the Agatston score of the aortic valve. Greater EFT volume is associated independently with preexisting first-degree AV block and new pacemaker implantation in patients undergoing TAVI. It may play a causative role in degenerative processes and the susceptibility of the AV conduction system.

## Introduction

Epicardial fat tissue (EFT) is the true visceral fat depot of the heart, accounting for approximately 20% of the total heart weight and covering nearly 80% of the heart’s surface [1–3]. Located between the myocardium and the visceral layer of the pericardium, EFT is in close contact with the epicardium without any borders or fascia and directly surrounds the coronary arteries [1, 3]. Physiologically, EFT has several cardioprotective functions. It protects the heart against mechanical stress and lower temperatures by serving as a physical buffer and generating heat [2]. It also serves as an energy source for the myocardium, providing free fatty acids in high-demand states and producing anti-inflammatory adipokines [2]. Conversely, EFT has unfavorable pro-inflammatory and pro-atherogenic properties, as it produces and releases cytokines such as interleukin (IL)-1, IL-6, IL-6sR, tumor necrosis factor (TNF)-α, and other bioactive molecules [4]. These agents are directly delivered into the adjacent myocardium and the coronary arteries via paracrine and vasocrine mechanisms [2]. Data strongly indicate a positive relationship between the extent of EFT and severity of CAD, CAD progression [5, 6], and coronary calcification [7]. Furthermore, EFT thickness has been shown to be associated with left ventricular (LV) mass and severe LV remodeling patterns in patients with high-grade aortic stenosis (AS) [8]. It is conceivable that other heart structures such as the aortic valve or the conduction system are also affected by the proinflammatory properties of EFT.

The calcific degeneration of the aortic valve is the most common cause of AS in the elderly over 70 years of age [9]. Calcification of the aortic valve is an ongoing, progressive, and complex process promoted by immunological, metabolic, and inflammatory factors that finally leads to AS [10]. However, the development of degenerative AS is still not fully understood.

Cardiac conduction abnormalities (CCAs) such as prolonged PR interval [e.g. first-degree atrioventricular (AV) block], right bundle branch block (RBBB), or left bundle branch block (LBBB) can have numerous etiologies, including ischemic or non-ischemic heart disease, acute myocardial infarction, infections, drugs/medication, and electrolyte abnormalities, but in the elderly fibrotic changes in the conduction system are thought to be the most common cause [11–14]. While the pathophysiology of ischemic, infectious, or infiltrative diseases leading to conduction abnormalities has been largely elucidated, the “degenerative” processes leading to alterations in the conduction system and subsequent development of conduction disorders, especially in older patients, are less understood and are still under investigation.

The aim of the present study was to investigate the association of EFT volume with preexisting conduction disturbances such as first-degree AV block, RBBB, and LBBB and the need for new pacemaker implantation after transcatheter aortic valve implantation (TAVI). Furthermore, a possible link between the calcium load of the aortic valve and EFT volume was examined. Our hypothesis is that proinflammatory characteristics of EFT negatively affect the conduction system by promoting conduction disturbances and aortic valve calcification.

## Methods

### Study cohort

The study population of this retrospective study consisted of all patients with high-grade AS who underwent a TAVI procedure between January 2016 and August 2017 (n = 657). Patients with a CT scan of insufficient quality (e.g. breathing artifacts or cropped heart borders) were excluded (n = 97). The final study cohort comprised 560 patients. The diagnosis of severe AS was made according to the established guideline recommendations [15]: a mean transvalvular gradient over the aortic valve of ≥ 40 mmHg or a valve area < 1 cm^2^ as calculated with the continuity equation or as measured by planimetry in transthoracic and/or transesophageal echocardiography.

The study was conducted in adherence to the Declaration of Helsinki and was approved by the Ethics Committee of the Justus-Liebig University of Giessen, Germany.

### Multidetector computed tomography (MDCT) analysis

Electrocardiogram-gated MDCT examinations were performed with a 128-slice or a 384-slice dual-source scanner (SOMATOM® Definition or Force; Siemens Healthineers, Forchheim, Germany) as previously described [16]. Reconstructions were carried out using a cardiac-gated B26f or I26f algorithm with a slice thickness of 0.6 mm in systole at 35% and in diastole at 70% of the cardiac cycle. The calcium score of the aortic valve was quantified using the Agatston method in non-contrast-enhanced scans of the aortic root with a threshold of 130 HU. The region of interest included the aortic valve and adjacent calcium deposits within the LV outflow tract. Regions that were incorrectly denoted as valvular calcium were cropped manually. Measurements of the aortic valve calcium score were performed offline on a dedicated workstation (Syn.govia, Siemens Healthineers, Forchheim, Germany).

### Measurement of EFT volume

**Fig. 1:**
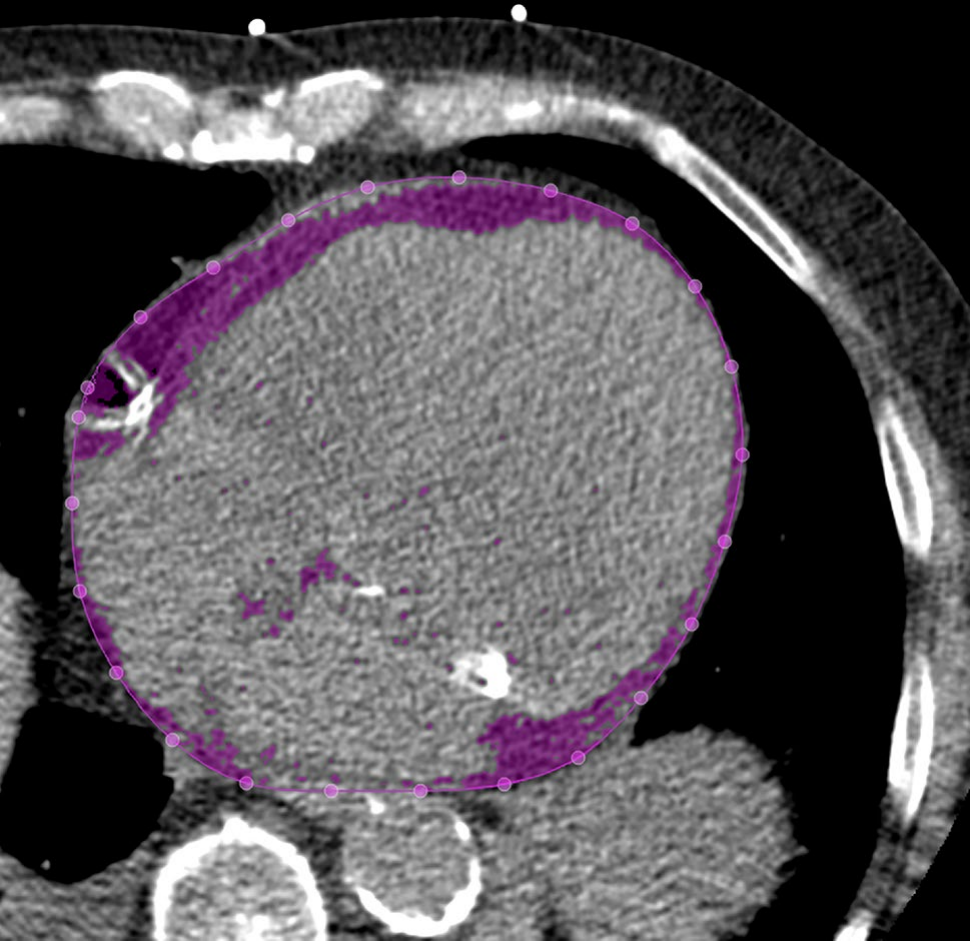
Example of an axial CT slice of the heart, with EFT shown in purple after automatic tracing of the pericardial border by the fully automated QFAT software. *CT* computed tomography, *EFT* epicardial fat tissue

EFT volume was quantified by using an established fully automated deep-learning algorithm that is a component of research software (QFAT V2.0, Cedars-Sinai Medical Center, Los Angeles, CA, USA) that has been validated and tested in a large multicenter study [17]. For EFT measurements the bifurcation of the pulmonary trunk was used as the upper boundary and the most inferior slice with any portion of the heart was used as the lower limit, as recommended by Dey et al. [18] When tracings of the pericardial border were insufficient due to limited image quality the tracings were manually adjusted. EFT volume was given in cm^3^. An example of EFT volume measurement is depicted in Fig. 1.

### ECG analysis

Twelve-lead ECGs obtained on admission prior to the TAVI procedure were used for measurements of PQ/PR, QRS, and QT intervals. Measurements of the PQ/PR interval were performed in lead II at the onset of the P wave to the beginning of the QRS segment. QRS and QT intervals were scaled in the precordial leads. The QT interval was defined as the time from the beginning of the QRS complex to the termination of the T wave. QTcorrected (QTc) was calculated by using the Bazett formula: QTc = QT/$\sqrt{RR}$. All measurements were performed manually by a single observer. ECGs with pacemaker activity or with distorted tracings were excluded from analysis.

### Statistical analysis

Continuous variables are presented as mean with standard deviation (SD) or as median with interquartile range (IQR), as appropriate. Categorical variables are given as numbers and percentages. The presence of a normal distribution pattern was tested using the Kolmogorov–Smirnov test. The Mann–Whitney-U test was used for comparison of continuous variables. For comparison of categorical variables, the Chi-squared test was applied. The Spearman Rho correlation was used to test for a link between EFT volume and different metric parameters such as body mass index (BMI), body surface area (BSA), age, Agatston score of the aortic valve, PQ/PR, QRS, and QT/QTc intervals. Univariate and multivariable logistic regression analysis was applied to analyze the association between EFT volume and conduction abnormalities and the need for new permanent pacemaker (PPM) implantation.

Variables with a p-value < 0.05 were included in multivariable logistic regression analysis. Significance was assumed when a two-sided p-value < 0.05 was determined, indicating that the null hypothesis was rejected. SPSS Version 22.0 (IBM, Armonk, New York, USA) was used for all statistical analyses.

## Results

### Patient characteristics

A total of 560 patients (54.3% female) with a median age of 82 years [IQR 79–86] were included in the analysis. The median EFT volume was 129.5 ml [IQR 94–170], and the median BSA-indexed EFT volume was 68.6 ml/m^2^. The median BMI was 26.4 [IQR 23.7–29.6] and the median Agatston score of the aortic valve was 2510 AU [IQR 1790–3475]. Preexisting first-degree AV block, RBBB, and LBBB were present in 17.0%, 10.4%, and 10.2% of patients, respectively. After TAVI, a new PPM was implanted in 11.8% (66/560) of patients. Table 1 depicts the baseline characteristics of the cohort.

**Table 1:** Baseline characteristics

Clinical characteristics	Total cohort n = 560
Age (years)	82 [79–86]
Sex, female	304 (54.3)
Body mass index (kg/m^2^)	26.4 [23.7–29.6]
Body surface area (m^2^)	1.85 [1.73–2.0]
Cardiovascular risk factors	
Hypertension	526 (93.9)
Diabetes mellitus	169 (30.2)
Hyperlipidemia	174 (31.1)
Echocardiographic variables	
LV ejection fraction (%)	65 [55–65]
Mean aortic transvalvular gradient (mmHg)	42 [32–52]
Aortic valve area (cm^2^)	0.7 [0.6–0.8]
ECG abnormalities	
Atrial fibrillation	236 (42.1)
AV block I	95 (17.0)
RBBB	58 (10.4)
LBBB	57 (10.2)
Cardiovascular disorders	
Coronary artery disease	346 (61.8)
Previous MI	78 (13.9)
Prior CABG	73 (13)
Peripheral artery disease	88 (15.7)
CT characteristics	
EFT volume (ml)	129.5 [94–170]
Agatston aortic valve score	2509.5 [1790–3474.5]

Values denote number (%) or median [interquartile range]

*LV* left ventricle, *ECG* electrocardiogram, *AV block* atrioventricular block, *RBBB* right bundle branch block, *LBBB* left bundle branch block, *MI* myocardial infarction, *CABG* coronary artery bypass graft, *CT* computed tomography, *EFT* epicardial fat tissue

### Correlation analysis of EFT with various variables

**Fig. 2:**
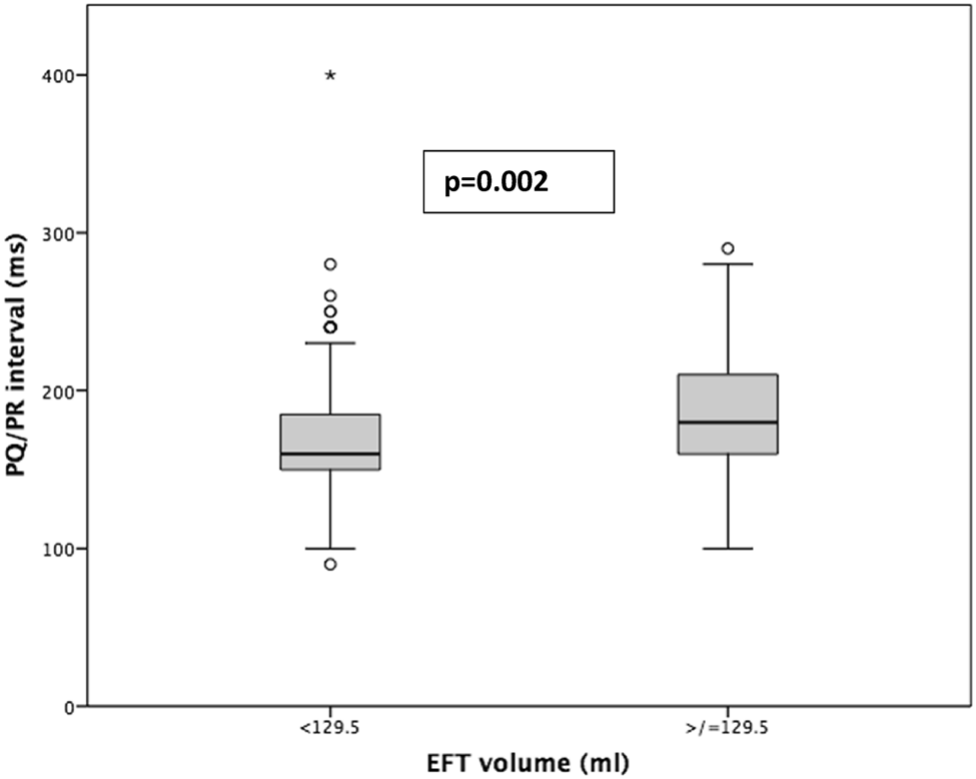
Comparison of dichotomized EFT volumes according to the median value (129.5 ml) in relation to PQ/PR interval. *EFT* epicardial fat tissue

EFT volume correlated moderately well with BMI and BSA (r = 0.48 and 0.54, respectively; both *p* < 0.001), whereas a weak inverse correlation was found for age (r =  − 0.11; *p* = 0.007). There was no correlation of EFT volume with the Agatston score of the aortic valve (r = 0.034; *p* = 0.43). Correlation analysis of ECG parameters showed a positive correlation of EFT volume and PQ/PR interval (r = 0.190; *p* < 0.001), but not for QRS, QT, or QTc intervals (Table 2). Figure 2 shows the differences of PQ/PR time in relation to EFT volume according to the median value.

**Table 2:** Correlation analysis of EFT volume with various parameters

	Spearman correlation coefficient r_s_	p-value
Age	− 0.115	***0.007***
BMI	0.475	**<** ***0.001***
BSA	0.541	**<** ***0.001***
Agatston score	0.340	0.430
PQ/PR interval (ms)	0.190	**<** ***0.001***
QRS interval (ms)	0.079	0.085
QT interval (ms)	0.029	0.522
QTc interval (ms)	0.042	0.365

Bold/italic p-values denote statistical significance at the level below 0.05

*EFT* epicardial fat tissue, *BMI* body mass index, *BSA* body surface area

### Association between EFT volume and conduction abnormalities and new PPM after TAVI

In univariate logistic regression analysis EFT volume was associated with the presence of preexisting first-degree AV block (OR 1.006 [95% CI 1.002–1.011]; *p* = 0.004) but not with RBBB or LBBB (OR 1.003 [95% CI 0.999–1.008]; *p* = 0.123 and OR 0.998 [95% CI 0.993–1.003]; *p* = 0.399). In multivariable regression analysis, after adjustment for sex, diabetes, CAD, and prior myocardial infarction, EFT volume remained independently associated with first-degree AV block (Table 3).

**Table 3:** Clinical determinants of first degree AV block

	Univariate OR [95% CI]	p-value	Multivariable OR [95% CI]	p-value
Age	0.996 (0.957–1.036)	0.835	–	–
Sex, male	2.23 (1.417–3.509)	***0.001***	1.609 (0.965–2.683)	0.068
Hypertension	1.569 (0.54–4.562)	0.408	–	–
Diabetes mellitus	1.7 (1.076–2.686)	***0.023***	1.551 (0.94–2.56)	0.086
CAD	2.5 (1.489–4.199)	***0.001***	2.508 (1.361–4.623)	***0.003***
Prior MI	2.031 (1.162–3.55)	***0.013***	1.488 (0.788–2.811)	0.22
Prior CABG	1.743 (0.971–3.129)	0.063	–	–
LV ejection fraction	0.997 (0.979–1.015)	0.715	–	–
EFT volume	1.006 (1.002–1.011)	***0.004***	1.006 (1.002–1.01)	***0.006***

Bold/italic p-values denote statistical significance at the level below 0.05

*CI* confidence interval, *OR* odds ratio, *CAD* coronary artery disease, *MI* myocardial infarction, *CABG* coronary artery bypass graft, *LV* left ventricle, *EFT* epicardial fat tissue

In univariate analysis, EFT volume was associated with the need for new PPM implantation after TAVI (OR 1.006 [95% CI 1.002–1.011; *p* = 0.004] and remained an independent predictor of new PPM after adjustment for sex, first-degree AV block, and RBBB (Table 4).

**Table 4:** Clinical determinants of new PPM implantation after TAVI

	Univariate OR [95% CI]	p-value	Multivariable OR [95% CI]	p-value
Age	1.034 (0.984–1.087)	0.182	–	–
Sex, male	1.787 (1.054–3.028)	***0.031***	1.179 (0.662–2.099)	0.576
LV ejection fraction	1.004 (0.981–1.027)	0.744	–	–
First degree AV block	2.567 (1.434–4.596)	***0.002***	2.205 (1.173–4.147)	***0.014***
RBBB	6.439 (3.463–11.973)	** < *0.001***	6.099 (3.208–11.595)	** < *0.001***
LBBB	0.562 (0.195–1.62)	0.286	–	–
EFT volume	1.006 (1.002–1.011)	***0.004***	1.005 (1.0–1.01)	***0.035***
Implantation depth, NCC	1.093 (0.991–1.205)	0.075	–	–
THV with high PPM risk^a^	2.005 (0.94–4.277)	0.072	–	–

Bold/italic p-values denote statistical significance at the level below 0.05

*CI* confidence interval, *OR* odds ratio, *LV* left ventricle, *AV block* atrioventricular block, *RBBB* right bundle branch block, *LBBB* left bundle branch block, *EFT* epicardial fat tissue, *NCC* non-coronary cusp, *THV* transcatheter heart valve, *PPM* permanent pacemaker

^a^High PPM risk prosthesis: Portico THV (Abbott, Chicago, Illinois, USA), CoreValve Evolut R (Dublin, Ireland), Lotus (Boston Scientific, Marlborough, Massachusetts, USA)

## Discussion

In this study we demonstrated that increased EFT volume is associated with preexisting first-degree AV block and the need for new PPM implantation in patients undergoing TAVI for severe AS. There was no association between EFT volume and preexisting RBBB, LBBB, or the amount of aortic valve calcification. To our knowledge this is the first study to evaluate the potential role of EFT in conduction disturbances in patients planned for TAVI and the first to investigate a potential association of EFT with the need for new PPM implantation after the TAVI procedure.

While vast amounts of data show strong evidence of a distinct positive association between EFT amount and both the incidence and the recurrence of atrial fibrillation [19–21], only a few studies have investigated the role of EFT in CCAs. Hung et al. analyzed the association between EFT amount and PR prolongation in a cohort of 287 patients referred for coronary CT analysis for exclusion of CAD [22]. The authors found that higher EFT volume was independently associated with PR prolongation after adjustment for the cardiovascular risk factors, age, sex, and BMI. ROC analysis revealed a c-statistic of 0.718 (95% CI 1.003–1.022; p = 0.0012) for EFT volume to predict PR prolongation and a threshold value of > 144.4 cm^3^. Likewise, Jhuo et al. observed an increased PR interval in patients with higher amounts of epicardial fat; the incidence of inter-atrial block defined as P wave prolongation ≥ 120 ms and biphasic P wave in the inferior leads of a 12-lead ECG were significantly associated with elevated epicardial fat volume [23]. In neither of these studies, however, was an association of EFT volume and QT and/or QTc interval length observed, which is in line with our findings. While the incidence of RBBB and LBBB in relation to EFT volume was not examined in either of the two studies, Hung et al. analyzed the QRS interval length in relation to higher EFT amounts but did not find an association [22]. This was also true for our study, which is consistent with the finding that EFT volume was not associated with RBBB or LBBB. Interestingly, EFT volume remained an independent predictor of new PPM implantation after TAVI along with first-degree AV block and RBBB, both well-known risk factors for complete heart block and subsequent need for PPM after TAVI [24]. Mechanical stress on the conduction system arising from radial forces of the transcatheter valves is known to play a crucial role in complete heart block in patients with preexisting first-degree AV block and RBBB [25], but the causative role of EFT in that scenario is less clear. We can only speculate that the greater pro-inflammatory capacity of higher amounts of EFT make the conduction system of the interventricular septum more “vulnerable” and potentially prolong the healing process in the surrounding tissues, thus increasing the risk of conduction disturbances and the need for PPM after TAVI.

In order to transfer our new finding of a predictive role of EFT in the need for PPM implantation after TAVI to the real-world clinical setting, clinicians analyzing pre-TAVI CT images may be advised to perform a measurement of EFT volume in order to further evaluate the potential risk of PPM implantation after TAVI. As CT imaging before TAVI is mandatory for the planning of the TAVI procedure, no additional examinations would be required and no further costs would arise. To confirm the clinical applicability of our findings, however, further studies are warranted.

Several previous studies were able to demonstrate a clear relationship of EFT amount and the extent and progression of CAD in terms of the degree of coronary artery stenosis, the incidence of “high-risk” plaques, and the extent of coronary artery calcification [26, 27]. It is postulated that the pathophysiological mechanism involves the release of pro-inflammatory and pro-atherogenic mediators from EFT, and higher EFT amounts may contribute to the development of atherosclerotic changes in the adjacent coronary arteries and subsequently to CAD [28].

As inflammatory processes are also assumed to play a crucial role in the development of degenerative aortic valve stenosis [9], a potential association of EFT with degenerative AS is plausible. A few studies have provided evidence of a positive relationship between the amount of EFT and the development of AS. Parisi et al. found an increased EFT thickness in patients referred for surgical aortic valve replacement due to high-grade AS compared with matched controls without AS [29]. Furthermore, greater EFT thickness significantly correlated with elevated serum levels of the pro-inflammatory cytokines IL-6, TNF-α, IL-1β, and MCP-1 [30]. In our study, there was no positive correlation between EFT and the Agatston score of the aortic valve. This would appear to contradict the findings of other studies: in 225 patients undergoing coronary angiography due to new-onset angina, Nabati et al. found higher amounts of aortic valve sclerosis expressed via an aortic valve sclerosis score index in patients with an echocardiographic EFT thickness ≥ 7 mm [30]. In addition, Mahabadi et al. observed a greater EFT thickness measured via echocardiography in patients with severe AS compared with patients without AS [31]. These findings were still significant after adjustment for age, sex, and cardiovascular risk factors. In our study, however, all patients in our cohort had degenerative high-grade AS, and we examined the correlation of EFT volume with the extent of aortic valve calcification reflected by the Agatston score, an entirely different approach. Comparison with a non-AS cohort would have been useful to explore possible effects of differences in EFT volumes, but this was beyond the scope of our study. One can speculate that once the degree of stenosis has reached a certain level of severity, there are no longer any major differences in EFT amounts between individuals. Moreover, not only calcification but also fibrotic changes in the aortic valve contribute to its narrowing, which are not captured by the Agatston score. Alternatively, it is conceivable that patients who finally develop severe AS already have higher amounts of EFT from the beginning of development of AS compared with those individuals who will not develop AS, and there may be no further changes in EFT volume during AS progression. This hypothesis can only be confirmed in large patient cohorts who are observed over many years with repeated measurements of the EFT thickness/volume and precise evaluation of the aortic valve and its degenerative process over time. Notably, aortic valve calcification is a complex and multifactorial process, and there are certainly further mechanisms, including endothelial dysfunction, mechanical stress, lipid infiltration, and oxidative stress that contribute to the calcification process of the aortic valve [9].

Our study has several limitations. First, the sample size of 560 patients is rather small, although among studies that investigated the role of EFT in different cardiac pathologies in the special subset of patients with AS, ours analyzed the highest number of patients. Secondly, we did not provide any information on the patients’ medication before TAVI, especially potential medications influencing cardiac rhythm and heart rate. Thirdly, although our findings demonstrate an association between EFT and conduction disturbances or increased PPM rate after TAVI, the causative role of EFT in that setting remains to be elucidated, and further studies are warranted to confirm our findings.

## Conclusion

Higher EFT volumes are independently associated with preexisting first-degree AV block and the need for new PPM implantation after TAVI. Further studies are needed to elucidate the causative role of EFT in these findings.

## References

[1] Iacobellis G, Bianco AC (2011). Epicardial adipose tissue: emerging physiological, pathophysiological and clinical features. Trends Endocrinol Metab.

[2] Villasante Fricke AC, Iacobellis G (2019). Epicardial adipose tissue: clinical biomarker of cardio-metabolic risk. Int J Mol Sci.

[3] Rabkin SW (2007). Epicardial fat: properties, function and relationship to obesity. Obes Rev.

[4] Mazurek T, Zhang L, Zalewski A, Mannion JD, Diehl JT, Arafat H, Sarov-Blat L, O'Brien S, Keiper EA, Johnson AG, Martin J, Goldstein BJ, Shi Y (2003). Human epicardial adipose tissue is a source of inflammatory mediators. Circulation.

[5] Mancio J, Azevedo D, Saraiva F, Azevedo AI, Pires-Morais G, Leite-Moreira A, Falcao-Pires I, Lunet N, Bettencourt N (2018). Epicardial adipose tissue volume assessed by computed tomography and coronary artery disease: a systematic review and meta-analysis. Eur Heart J Cardiovasc Imaging.

[6] Conceicao G, Martins D, Miranda IM, Leite-Moreira AF, Vitorino R, Falcao-Pires I (2020). Unraveling the role of epicardial adipose tissue in coronary artery disease: partners in crime?. Int J Mol Sci.

[7] Rosito GA, Massaro JM, Hoffmann U, Ruberg FL, Mahabadi AA, Vasan RS, O'Donnell CJ, Fox CS (2008). Pericardial fat, visceral abdominal fat, cardiovascular disease risk factors, and vascular calcification in a community-based sample: the Framingham Heart Study. Circulation.

[8] Coisne A, Ninni S, Ortmans S, Davin L, Kasprzak K, Longere B, Seunes C, Coppin A, Mouton S, Ridon H, Klein C, Noirot-Cosson B, Staels B, Lancellotti P, Montaigne D, Pontana F (2019). Epicardial fat amount is associated with the magnitude of left ventricular remodeling in aortic stenosis. Int J Cardiovasc Imaging.

[9] Conte M, Petraglia L, Campana P, Gerundo G, Caruso A, Grimaldi MG, Russo V, Attena E, Leosco D, Parisi V (2020). The role of inflammation and metabolic risk factors in the pathogenesis of calcific aortic valve stenosis. Aging Clin Exp Res.

[10] Cho KI, Sakuma I, Sohn IS, Jo SH, Koh KK (2018). Inflammatory and metabolic mechanisms underlying the calcific aortic valve disease. Atherosclerosis.

[11] Cheng SKM, Larson MG, McCabe EL, Newton-Cheh C, Levy D, Benjamin EJ, Vasan RS, Wang TJ (2009). Long-term outcomes in individuals with prolonged PR interval or first-degree atrioventricular block. JAMA.

[12] Oldroyd SH, Quintanilla Rodriguez B, Makaryus AN (2021). First degree heart block.

[13] Harkness WT, Hicks M (2021). Right bundle branch block.

[14] Tan NY, Witt CM, Oh JK, Cha Y-M (2020). Left bundle branch block. Circ Arrhythm Electrophysiol.

[15] Joint Task Force on the Management of Valvular Heart Disease of the European Society of Cardiology, European Association for Cardio-Thoracic Surgeons, Vahanian A, Alfieri O, Andreotti F, Antunes MJ, Baron-Esquivias G, Baumgartner H, Borger MA, Carrel TP, De Bonis M, Evangelista A, Falk V, Iung B, Lancellotti P, Pierard L, Price S, Schafers HJ, Schuler G, Stepinska J, Swedberg K, Takkenberg J, Von Oppell UO, Windecker S, Zamorano JL, Zembala M (2012) Guidelines on the management of valvular heart disease (version 2012). Eur Heart J 33(19):2451–2496.10.1093/eurheartj/ehs109 10.1093/eurheartj/ehs109 22922415

[16] Kim WK, Renker M, Rolf A, Fischer-Rasokat U, Wiedemeyer J, Doss M, Mollmann H, Walther T, Nef H, Hamm CW, Liebetrau C (2019). Annular versus supra-annular sizing for TAVI in bicuspid aortic valve stenosis. EuroIntervention.

[17] Commandeur F, Goeller M, Razipour A, Cadet S, Hell MM, Kwiecinski J, Chen X, Chang HJ, Marwan M, Achenbach S, Berman DS, Slomka PJ, Tamarappoo BK, Dey D (2019). Fully automated CT quantification of epicardial adipose tissue by deep learning: a multicenter study. Radiol Artif Intell.

[18] Dey D, Wong ND, Tamarappoo B, Nakazato R, Gransar H, Cheng VY, Ramesh A, Kakadiaris I, Germano G, Slomka PJ, Berman DS (2010). Computer-aided non-contrast CT-based quantification of pericardial and thoracic fat and their associations with coronary calcium and metabolic syndrome. Atherosclerosis.

[19] Al Chekakie MO, Welles CC, Metoyer R, Ibrahim A, Shapira AR, Cytron J, Santucci P, Wilber DJ, Akar JG (2010). Pericardial fat is independently associated with human atrial fibrillation. J Am Coll Cardiol.

[20] Batal O, Schoenhagen P, Shao M, Ayyad AE, Van Wagoner DR, Halliburton SS, Tchou PJ, Chung MK (2010). Left atrial epicardial adiposity and atrial fibrillation. Circ Arrhythm Electrophysiol.

[21] Mirolo A, Viart G, Savoure A, Godin B, Raitiere O, Eltchaninoff H, Anselme F (2019). Epicardial fat thickness predicts atrial fibrillation recurrence after a first pulmonary vein isolation procedure using a second-generation cryoballoon. Arch Cardiovasc Dis.

[22] Hung WC, Tang WH, Wang CP, Lu LF, Chung FM, Lu YC, Hsu CC, Tsai IT, Jhuo SJ, Lai WT, Lee YJ, Ya TH (2015). Increased epicardial adipose tissue volume is associated with PR interval prolongation. Clin Investig Med.

[23] Jhuo SJ, Hsieh TJ, Tang WH, Tsai WC, Lee KT, Yen HW, Lai WT (2018). The association of the amounts of epicardial fat, P wave duration, and PR interval in electrocardiogram. J Electrocardiol.

[24] Siontis GC, Juni P, Pilgrim T, Stortecky S, Bullesfeld L, Meier B, Wenaweser P, Windecker S (2014). Predictors of permanent pacemaker implantation in patients with severe aortic stenosis undergoing TAVR: a meta-analysis. J Am Coll Cardiol.

[25] Mangieri A, Montalto C, Pagnesi M, Lanzillo G, Demir O, Testa L, Colombo A, Latib A (2018). TAVI and post procedural cardiac conduction abnormalities. Front Cardiovasc Med.

[26] Rajani R, Shmilovich H, Nakazato R, Nakanishi R, Otaki Y, Cheng VY, Hayes SW, Thomson LE, Friedman JD, Slomka PJ, Min JK, Berman DS, Dey D (2013). Relationship of epicardial fat volume to coronary plaque, severe coronary stenosis, and high-risk coronary plaque features assessed by coronary CT angiography. J Cardiovasc Comput Tomogr.

[27] Yu W, Liu B, Zhang F, Wang J, Shao X, Yang X, Shi Y, Wang B, Xu Y, Wang Y (2021). Association of epicardial fat volume with increased risk of obstructive coronary artery disease in Chinese patients with suspected coronary artery disease. J Am Heart Assoc.

[28] Tanaka K, Sata M (2018). Roles of perivascular adipose tissue in the pathogenesis of atherosclerosis. Front Physiol.

[29] Parisi V, Rengo G, Pagano G, D'Esposito V, Passaretti F, Caruso A, Grimaldi MG, Lonobile T, Baldascino F, De Bellis A, Formisano P, Ferrara N, Leosco D (2015). Epicardial adipose tissue has an increased thickness and is a source of inflammatory mediators in patients with calcific aortic stenosis. Int J Cardiol.

[30] Nabati M, Favaedi M, Kheirgoo M, Yazdani J, Dabirian M (2018). Correlation between epicardial fat thickness and aortic valve sclerosis. Asian Cardiovasc Thorac Ann.

[31] Mahabadi AAKH, Dykun I, Balcer B, Kahlert P, Rassaf T (2017). Epicardial adipose tissue thickness independently predicts severe aortic valve stenosis. J Heart Valve Dis.

